# Cancer ego-system in glioma: an iron-replenishing niche network systemically self-organized by cancer stem cells

**DOI:** 10.1186/s41232-022-00240-8

**Published:** 2022-11-30

**Authors:** Kouichi Tabu, Tetsuya Taga

**Affiliations:** Department of Stem Cell Regulation, Medical Research Institute, Tokyo Medical and Dental University (TMDU), Tokyo, 113-8510 Japan

**Keywords:** Cancer ecosystem, Cancer stem cell, Niche, Glioma, Tumor-associated macrophage, Erythropoiesis, Erythrophagocytosis, Iron

## Abstract

For all living organisms, the adaptation to outside environments is an essential determinant to survive natural and artificial selections and to sustain the whole ecosystem intact with functional biodiversity. Likewise, cancer cells have similar characteristics that evade not only stresses from the host-internal innate and adaptive immune systems but also those from host-externally administered therapeutic interventions. Such selfish characteristics of cancer cells lead to the formation of cancerous ecosystem with a wide variety of phenotypic heterogeneity, which should be called cancer “egosystem” from the host point of view. Recently increasing evidence demonstrates that cancer stem cells (CSCs) are responsible for this cancer egosystem by effectively exploiting host inflammatory and hematopoietic cells and thereby reconstructing their own advantageous niches, which may well be a driving force in cancer recurrence. CSCs are further likely to render multiple niches mutually interconnected and cooperating as a network to support back CSCs themselves. Here, we summarize a recently identified iron-replenishing niche network self-organized by glioma CSCs (GSCs) through remote regulation of host myeloid and erythroid lineage cells. GSCs recruit bone marrow (BM)-derived inflammatory monocytes into tumor parenchyma, facilitate their differentiation into macrophages (Mφs) and skew their polarization into pro-tumoral phenotype, i.e., tumor-associated Mφs (TAMs). Meanwhile, GSCs distantly enhance erythropoiesis in host hematopoietic organs like BM and spleen potentially by secreting some soluble mediators that maintain continuous supply of erythrocytes within tumors. In addition, as normal red pulp Mφs (RPMs) under steady state conditions in spleen recycle iron by phagocytosing the aged or damaged erythrocytes (a/dECs) and release it in time of need, TAMs at least in gliomas phagocytose the hemorrhaged erythrocytes within tumors and potentially serve as a source of iron, an important nutrient indispensable to GSC survival and glioma progression. Taken together, these studies provide the substantial evidence that CSCs have a unique strategy to orchestrate multiple niches as an ecosystem that threatens the host living, which in this sense must be an egosystem. Targeting such an adaptive subpopulation of CSCs could achieve drastic disturbance of the CSC niches and subsequent extinction of malignant neoplasms.

## Background

All organisms living on this Earth have acquired the inheritable genetic and epigenetic variations through the past millions of years and have adapted to natural selections by various environmental changes such as climate change and volcanic eruption, resulting in a sustainable ecosystem with functional diversities. In such an ecological view, cancers have also been considered as malignant neoplasm forming their own advantageous ecosystem (cancer ecosystem) inside a tumor [1]. In 1976, Nowell provided the evolutional views into tumor expansion as a stochastic process of genetic instability and natural selection (clonal evolution model) [2], in which individual cells may asynchronously acquire different types of gene alterations and once a mutation confers the advantages in malignant progression, more proliferative and adaptive clones dominantly expand to occupy the entire tumors. Subsequently, Bonnet and Dick in 1997 appended there an unprecedented perspective of stem cell hierarchy (CSC model), in which only CSCs with stem-like properties inherit the tumorigenic potential to recapitulate a heterogeneous cancer population of their identical copy (self-renewal) and daughter progenies terminally growth-arrested (differentiation) [3]. Consistent with the former model, CSCs are highly adaptive to the stresses by host-internal events like immune surveillance; e.g., programmed cell death-1 ligand-1 (PD-L1) (a negative regulator of T cells) is upregulated at the invasive edge, thereby tumor cells suppressing T cell activation and survival [4], natural killer group 2D (NKG2D) ligand (an activator of NK cells) is downregulated by tumor hypoxia enabling tumor cells to evade from recognition by NK cells [5] and major histocompatibility complex (MHC)-I (a presenter of foreign antigens to T cells) is defective in CSCs, thereby CSCs disturbing the T cells’ effector functions [6], and therefore the two models above are not necessarily mutually exclusive. It must be further noteworthy that the CSC concept explains the adaptation not only to the stresses from the host-internal innate and acquired immune responses but also to those from host-externally administered therapeutic interventions, e.g., CSCs are escapable from surgical resections due to their high invasiveness and metastatic properties [7], and resistant to chemical- and radiation-induced cell death due to their high abilities that expel xenobiotics [8], repair DNA damage [9], and scavenge reactive oxygen species [10]. More importantly, it has become apparent that CSCs have the nature that forms their own self-advantageous niche microenvironment by in part taking over and controlling remotely the host inflammatory and hematopoietic cells [11, 12, 13]. This type of niche can be considered as a cancer ecosystem but appears to be a cancer egosytem from the viewpoint of cancer-bearing host. To facilitate the development of innovative therapies targeting cancer egosystem and their developmental process, we review here recent advance in understanding of how glioma CSCs (GSCs) systemically organize multiple niches in the host living, especially by focusing on a recently identified iron replenishing network self-constructed by GSCs in brain by exploiting peripheral myeloid and erythroid lineage cells.

### BM-derived monocytes/macrophages, major components of glioma microenvironments

Gliomas are the most frequent primary brain tumors characterized by rapid and invasive growth into the brain parenchyma, enabling them to escape from surgical resection. The most malignant glioma, also known as glioblastoma multiform (GBM), typically recurs within 1 year despite the aggressive treatments with anti-glioma drug temozolomide and radiotherapy and is therefore one of the intractable cancers with the poorest prognosis; 5-year survival rate is less than 10% [14].

As well as other cancers, GBM microenvironments are composed of various non-neoplastic cells of host origins, which includes vascular endothelial cells, immune cells derived from peripheral blood, and resident brain cells [15], forming a complex network of niche cells producing a wide variety of cytokines and chemokines that support tumor cell growth [16]. Histologically and experimentally, major components of immune cells in GBM tissues (more than 85%) has been identified as monocytes and Mφs [17, 18], strongly supporting dominant contribution of myeloid lineage cells to glioma progression [19]. Recent large-scale in silico analysis using TCGA transcriptome data from over 500 primary and recurrent GBM specimens has also quite reliably provided the evidence that among various lymphoid and myeloid lineage cells, M2 (anti-inflammatory)-, followed by M0 (resting)-Mφs and monocytes in this order, are the most abundant immune cells infiltrating into GBM microenvironments [12]. Convincingly, the infiltrations of CD8^+^ cytotoxic T cells and activated NK cells are significantly decreased in primary GBMs. It is also of interest that while these proportions are relatively similar between primary and recurrent GBMs, M2-Mφs are not increased and CD8^+^ T cells are not decreased in the recurrent cases, suggesting that dramatic reconstruction of immune cells composing microenvironments occur in the context of GBM recurrence. It must be of note that almost all of currently generalized concepts have been established from surgically resectable primary GBM specimens, i.e., from cells not responsible for recurrence, when therapeutic strategies to prevent GBM recurrence would be developed.

In human gliomas, TAMs are commonly identified by the expression of a broad monocyte lineage marker CD68 and M2-specific Mφ markers CD163 and CD204 [20, 21]. However, since not only CD68 and CD163 but also CD11b and Iba1 are all commonly expressed in all Mφ-like cells including resident microglias [22], some researchers use the term TAMs as the abbreviation of “tumor-associated Mφs/microglias” [23]. To resolve this issue, recent genetically engineered fate-mapping study by Chen et al. identified F4/80 as a marker to distinguish tumor-infiltrating Mφs from resident microglia, monocytes and neutrophils, accounting for more than 80% of all CD45^+^CD11b^+^ myeloid lineage cells in GBM tissues [24]. By taking advantage of fluorescent reporter system using *Cx3cr1* and *Ccr2* gene promoters, they further clarified that CX3CR1^-^CCR2^high^ inflammatory monocytes (iMo) from BM, even not CX3CR1^high^CCR2^-^ circulating ones infiltrate into GBM tissues, where they differentiate into CX3CR1^high^CCR2^low^ Mφs and CX3CR1^high^CCR2^-^ microglia-like cells, accounting for most of myeloid cells. Interestingly, the latest outstanding approach using high-dimensional single-cell profiling (CyTOF) deeply analyzed heterogenous composition of myeloid cells in brain tumor microenvironment (TME) at the single-cell level, and provided the conclusive evidence that TAM composition in gliomas is determined by isocitrate dehydrogenase (IDH) gene mutation; TME of relatively benign IDH1^mut^ gliomas dominantly contain resident microglia and few Mo-derived Mφs (MDMs), while more aggressive IDH^wt^ gliomas are characterized by the elevated infiltration of a large number of MDMs [25]. These studies clearly illustrate the possibility that genetic backgrounds of tumor cells and niche cells of origin within TME may fully define the overall tumor identities in glioma.

### Monocyte recruitment and TAM development by GSCs in GBMs

Consistent with the findings above, the conditioned media prepared from GSCs, not from non-GSCs effectively induced the differentiation of BM-derived monocytes into the F4/80^+^CD204^+^ Mφs, which exhibited protumoral effects when co-transplanted into mouse brain with GSCs [11]. In addition, the expression of *Ccl2* gene encoding the chemokine that binds with CCR2 and *Csf1* gene encoding the cytokine that induces the differentiation of monocytes into Mφs are highly upregulated in human high-grade gliomas [26, 27] and functionally contribute to glioma progression [24, 28, 29], totally supporting the idea that GSCs self-construct their own niche dominantly involving TAMs through remotely communicating with host CCR2^+^ BM-derived inflammatory monocytes, but rarely through expanding the resident microglias in brain.

It should be noted that at least three anatomically distinct niches, i.e., invasive, perinecrotic, and perivascular niches, have been identified in GBM tissues representing functional heterogeneity of GSCs within a tumor [15]. CD204^+^ TAMs are actually located in tumor-normal boundary, peri-palisading region and hemorrhagic area in glioma tissues [11], and notably in the peritumoral invasive region, a potentially unresectable area responsible for cancer recurrence, the number of microglias is larger rather than those of Mφs [24, 30]. However, abundant microglia in the brain tumor border does not necessarily account for less contributions of Mφs to glioma recurrence, as demonstrated that CD163+ Mφs form a GSC niche by interacting with oligodendrocyte progenitor cells [31] and that monocyte-derived Mφs are peripherally recruited and expanded after radiation [29], and therefore these spatiotemporal diversity of TAMs indicates that the timing of therapeutic interventions, e.g., pre-/post-surgical resection or radiation should be indispensable in considering for the successful targeting of TAMs and microglias in primary and recurrent GBMs.

Relating to this spatial issue, the phenotype of TAMs in gliomas remains still controversial, although TAMs are often defined as anti-inflammatory M2-Mφs (protumoral) and the ratios of M2-Mφs to monocyte lineages (CD163/CD68 and CD204/CD68) have been commonly used in histopathological diagnosis as important criteria highly associated with malignant grades in human gliomas [20]. However, recent studies with a unique viewpoint from cell death revealed that a fraction of GSC-derived necrotic particles morphologically and biochemically designed as autoschizis-like products (ALPs) spontaneously arising from glioma cells promoted the development of GSC-friendly M1-type CD204+ Mφs following their phagocytosis [12]. This study provides a broad significance not only in demonstrating a tumor beneficial role of necrosis for CSC-driven niche construction but also in proposing the presence of M1 marker-expressing Mφs with protumoral functions. This type of Mφs express both an M2 marker CD204 and M1 marker genes *Cd86*, *Tnfa*, and *Il12b*. Particularly, the proinflammatory cytokine IL-12, which plays a central role in the regulation of Th1 antitumoral immune responses, act as a niche factor that directly affects the growth of human GBM patient-derived GSCs. Supporting this observation, higher co-expression of *Il12a* and *Il12b* genes encoding p35 and p40 subunits of IL-12 has a big impact on the poorer prognosis in recurrent GBM patients. Finally, in silico flow cytometry analysis of transcriptome data definitely indicates that TAMs have a weak signature of M2-Mφs in primary GBMs, but display extremely heterogeneous signatures composed of multiple subtypes of Mφs (M0, M1, and M2) in recurrent ones; especially the expression of *Cd163* and *Cd204* genes have the strongest correlation with M1-Mφs among all myeloid lineage cells, indicating that a dramatic rearrangement of niche involving TAMs occurred in the phase of glioma recurrence and that CD204 is not a specific marker for M2-like TAMs but broadly expressed in all of resting and polarized TAMs. Contrary to the conventional view, the higher infiltration of M1-Mφs strongly associated with poor survival of patients with recurrent GBMs. Devising new therapeutics against not only M2- but also M1-like TAMs will be required for the inhibition of GSC-driven GBM recurrence.

### Enhanced erythropoiesis in GSC-mediated GBM formation

Erythroid lineages are also essential components of tumor microenvironments that play important roles in continuous supply of oxygen and iron into the tumors. Adult erythropoiesis mainly occurs in BM at the steady-state and maintains the homeostasis of circulating erythrocytes in body [32], whereas the extra-medullary erythropoiesis is triggered in spleen under the abnormal conditions including carcinogenesis; e.g., extramedullary erythropoiesis was observed in the spleen of breast tumor [33], fibrosarcoma and Lewis lung carcinoma [34], and so on. The latest studies by Aimaitijiang et al. have explored the impacts of GSCs on erythroid differentiation and show that the conditioned media from GBM patient-derived cells promote the maturation of TER119^low^ CD71^high^ proerythroblasts toward mature TER119^high^ CD71^low^ erythrocytes in vitro [13]. In addition, the profiling of erythroid lineages in hematopoietic organs of glioma-bearing mice showed a significant increase in the percentage and the absolute number of erythrocytes in BM and spleen without anemia, strongly suggesting that GSCs in brain remotely facilitate the medullar and extramedullar erythropoiesis in peripheral [13]. It should be reasonable that anemia is often observed in various cancer patients but is not the case with GBM patients.

Although the detail mechanisms how brain GSCs communicate with distant hematopoietic organs in peripheral have not been identified in their work, they provide some candidate genes of soluble factor(s) expressing in GSCs, which include BMP2, CSF2, and KITLG known to positively act on erythropoiesis, particularly the proliferation and differentiation of hematopoietic stem/progenitor cells [35, 36, 37]. For example, high expression of KITLG protein correlates with poor survival in GBM patients and its positivity is confirmed near to microvasculature along the tumor invasive border [38], where GSCs are engaged in GBM expansion and recurrence after surgical resection [39]. Erythropoietin (EPO) is also proposed as a major important factor mainly produced in kidney and has a central role in erythropoiesis, whose secretion is known to be elevated in multiple cancers [34] including human GBM tissues [40]. However, *Epo* gene is likely to be little expressed in GBM cells themselves but secreted by surrounding cells within GBM tissues [13, 40, 41]. Further investigations are needed to unravel whether GSC-derived soluble factor(s) trigger the EPO production in kidney to indirectly enhance the erythropoiesis and/or whether glioma microenvironment-derived EPO directly induces the erythropoiesis in BM and spleen. It is also of interest that CD71^+^ erythroid cells pre-exploded in spleen, infiltrate into tumor microenvironments and potently inhibit CD8^+^ and CD4^+^ T cell proliferation, differentiation, and cytotoxicity through secretion of multiple immunosuppressive factors [42, 43]. These works totally corroborate the idea that systemic cross-talks between brain GSCs and host hematopoietic organs in peripheral foster glioma progression.

### Erythrophagocytosis and iron recycling by TAMs

It is well-known that TAM markers CD163 and CD204 are functional molecules that scavenge hemoglobin and various kinds of macromolecules, suggesting the potential involvement of erythrophagocytosis and iron recycling by TAMs in cancer malignancies. Given that iron is little available from the food, all living organisms innately possess the abilities to recycle iron (approximately 90% of needs) to maintain erythropoiesis throughout the life [44]. Especially, RPMs in spleen are specialized for iron recycling with the abilities to phagocytose naturally aged and damaged erythrocytes (a/dECs), which strictly regulated not only by the interaction of phosphatidylserine (PS) externalized on a/dECs with T cell immunoglobulin and mucin domain containing 4 (TIM4) receptor expressing on RPMs (“eat me” signal) but also by the interaction of CD47 on young erythrocytes with signal regulatory protein α (SIRPα) receptor on RPMs (“don’t eat me” signal). After engulfmented by RPMs, RBCs are degraded into cellular components in phagolysosomes and heme is released and catabolized into CO, biliverdin and iron. Iron is subsequently stored with ferritin in cytoplasm and released into blood stream upon the increased demands of the body. On the other hand, CD169^+^ Mφs in BM (namely nurse Mφs), highly expressing the receptors for an iron-carrier transferrin (Tf), function as a niche for erythropoiesis called erythroblastic islands that supplies iron for heme/hemoglobin synthesis to erythroid progenitors. Surprisingly, GBMs also possess a similar iron-replenishing network composed of hemorrhaged erythrocytes and phagocytosing TAMs that maintains iron homeostasis within a tumor; i.e., immunohistochemical, DAB and Prussian blue staining of GSC-driven GBM tissues showed that at least a part of CD204^+^ TAMs phagocytose the hemorrhaged erythrocytes and store iron [11]. These erythrophagocytosing and iron-storing phenotypes of TAMs in glioma are definitely verified in vitro coculture experiments where F4/80^+^CD204^+^ Mφs induced by GSC-derived conditioned media more efficiently engulf erythrocytes than those induced by non-GSCs. It must be convincing that *Tf* gene is upregulated in non-GSCs and conversely its receptor *Tfrc* gene is upregulated in GSCs. Supporting these observations, a recent study of 21 adult GBM patients to investigate the correlation between radiology and pathology using quantitative susceptibility mapping (an MRI-based method for estimating the distribution of magnetic molecules such as iron) demonstrates a significant correlation between mean susceptibility and the ferritin positivity in tumor zones [45]. In addition, a large cohort study of 111 malignant astrocytic brain tumors (grade II–IV) with immunohistochemical staining clearly showed that not only high TfR1 expression level but also high ferritin-expressing microglial score are significantly correlated with short survival [46], strongly corroborating the presence of a TAM-mediated iron deposition and recycling network in glioma as an egoistic system by iron-demanding GSCs functionally contributing to glioma progression. Taken together, there is no doubt that GSCs have the consummate egotism that systemically constructs a cancer ecosystem by ingeniously possessing host hematopoiesis. Further investigation of this niche network must discover the detail mechanisms of when, where and how TAMs sense the demands of iron and supply it to GSCs in context of glioma progression, therapy resistance and recurrence.

## Conclusion

**Fig. 1:**
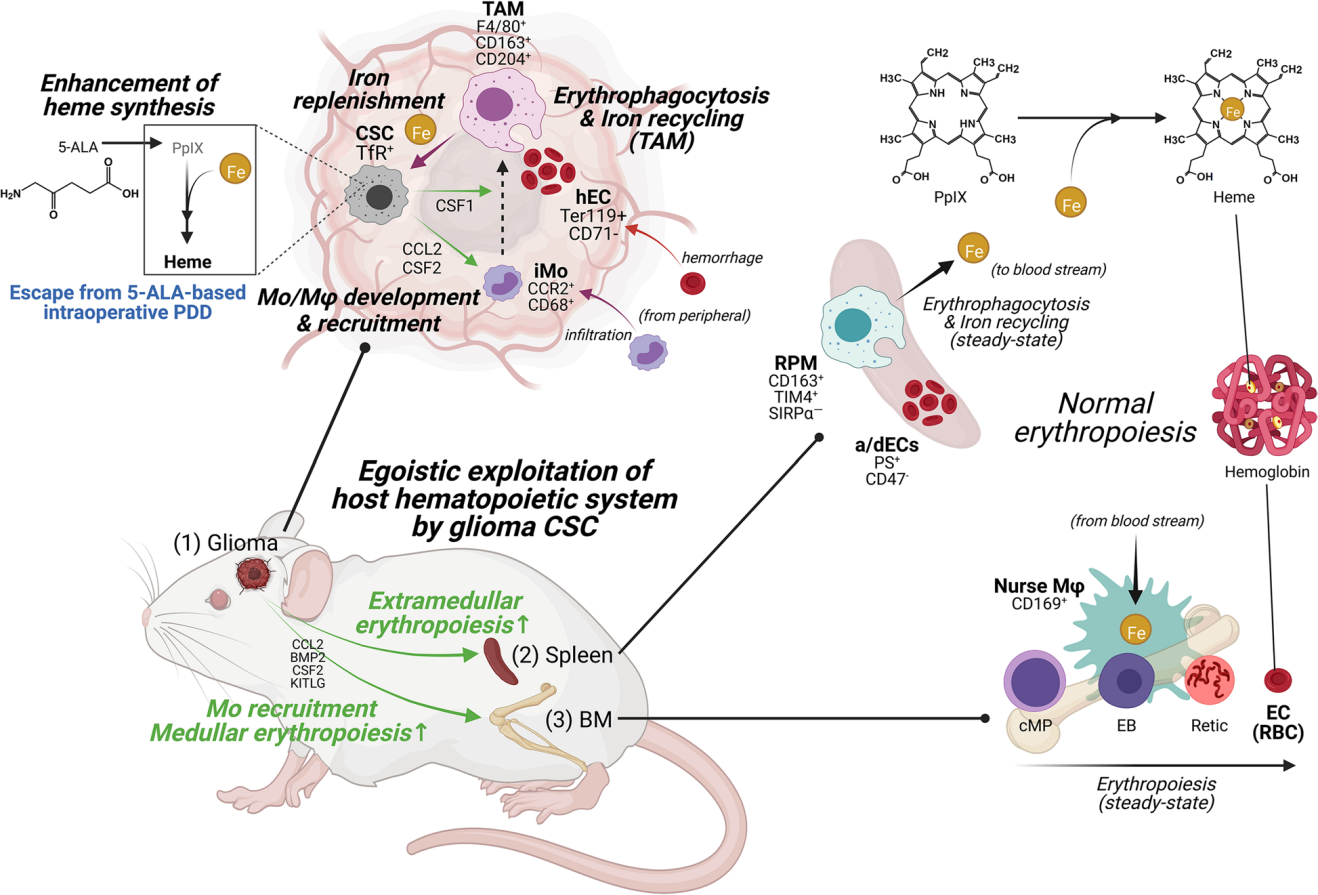
Systemic cancer ecosystem self-organized by CSCs in glioma-bearing mice. A functional niche network for CSCs to replenish iron, indispensable to GSC expansion themselves, is delineated by interconnecting GSC-induced TAM development in brain, and GSC-triggered enhanced erythropoiesis in peripheral. (1) Glioma: TfR^+^ GSCs recruit CCR2^+ ^CD68^+ ^inflammatory monocytes (iMo) from peripheral BM and induce F4/80^+ ^CD163^+ ^CD204^+^ tumor-associated macrophages (TAMs) through secreting some soluble factors such as CCL2 and CSF1/2 [Mo/Mφ development]. On the other hand, GSCs facilitate medullar and extramedullar erythropoiesis in peripheral hematopoietic organs, i.e., BM and spleen, potentially through secreting some soluble mediators. Collectively, Ter119^+^ hemorrhaged erythrocytes (hEC) infiltrating from peripheral are phagocytosed by the TAMs in brain and catabolized into iron [erythrophagocytosis and iron recycling]. Iron is stored in TAMs and potentially released upon the demands of GSCs highly expressing the receptor for the iron-carrier transferrin (Tf) [iron replenishment]. (2) Spleen: CD163^+ ^TIM4^+^ red pulp macrophages (RPMs) originally have the ability to engulf the aged and damaged erythrocytes (a/dECs) externalizing phosphatidylserine (PS) at the cell surface to recycle and release iron into blood stream at the time of needs [erythrophagocytosis and iron recycling]. (3) Bone marrow (BM): hematopoietic differentiation from common myeloid progenitors (cMP) to erythroblasts (EB), reticulocytes (Retic), and erythrocytes (EC) is enhanced in glioma-bearing mouse BM [erythropoiesis], potentially maintaining the influx of erythrocytes hemorrhaged into glioma tissues. In BM erythropoiesis, CD169^+^ nurse macrophages uptake the recycling iron from blood stream and supply it for EBs to synthesize hemoglobin from heme composed of protoporphyrin IX (PpIX) and iron. Such heme synthesis pathway is also elevated in iron-demanding TfR^+^ GSCs, causing them to escape from 5-ALA-based photodynamic diagnosis (PDD) during surgical operation

We have reviewed here a network of niche systemically self-organized by CSCs in glioma, which is extremely egoistic: i.e., (1) CSCs recruit inflammatory monocytes from host BM into brain and induce the differentiation into TAMs within tumor microenvironments, and simultaneously, (2) CSCs enhance the erythropoiesis in BM and spleen potentially to maintain the influx of erythrocytes hemorrhaged in tumor microenvironments, and finally (3) TAMs phagocytose the hemorrhaged erythrocytes and potentially function as an intratumoral source of iron (Fig. 1). Recent works have corroborated this scenario by the substantial evidence of strong demands for iron in CSCs; several iron chelators are trialed for clinical studies which include deferoxamine (for neuroblastomas and prostate cancers), and Triapine (for leukemia, renal cell carcinomas and head and neck squamous cell carcinomas) etc. It is noteworthy that CSCs are escapable from 5-aminolevulinic acid (5-ALA)-based fluorescence-guided photodynamic diagnosis/therapy due to their abnormal metabolism in iron-containing heme biosynthesis pathway [47]. Altogether, this review pronouncedly exemplify a systemic network of niches egoistically constructed by CSCs in host, so called cancer “ego-system”, where host myeloid and erythroid lineage cells are abnormally interconnected and collaborating for maintaining iron homeostasis within a tumor, supporting the ecological concept for cancer therapeutics that targeting adaptive (niche-constructing) subpopulation of CSCs could yield drastic disturbance of niche and then extinction of malignant neoplasm.

## Data Availability

Further information and requests for resources and reagents should be directed to the authors: Kouichi Tabu (k-tabu.scr@mri.tmd.ac.jp) and Tetsuya Taga (taga.scr@mri.tmd.ac.jp).

## Abbreviations

5-ALA: 5-aminolevulinic acid
a/dEC: Aged and damaged erythrocyte
BM: Bone marrow
CSC: Cancer stem cell
DAB: 3,3′-Diaminobenzidine
EPO: Erythropoietin
GBM: Glioblastoma multiform
GSC: Glioma stem cell
Mφ: Macrophage
MRI: Magnetic resonance imaging
NK: Natural killer (NK)
PDD: Photodynamic diagnosis
RBC: Red blood cell
RPM: Red pulp macrophage
TAM: Tumor-associated Mφ
Tf: Transferrin
TfR: Transferrin receptor
TME: Tumor microenvironment
